# The Psychology of Sustainability and Sustainable Development: Advancing Decent Work, Inclusivity, and Positive Strength-Based Primary Preventive Interventions for Vulnerable Workers

**DOI:** 10.3389/fpsyg.2021.718354

**Published:** 2021-07-27

**Authors:** Annamaria Di Fabio, Andrea Svicher

**Affiliations:** Department of Education, Languages, Intercultures, Literatures and Psychology (Psychology Section), University of Florence, Florence, Italy

**Keywords:** psychology of sustainability, sustainable development, decent work, inclusivity, vulnerable workers, healthy organizations, strength-based primary prevention perspective

## Abstract

This study discusses the contribution of the psychology of sustainability and sustainable development to the wellbeing of vulnerable workers. The psychology of sustainability and sustainable development is a current area of the research study in the field of sustainability science. It deals with sustainability as a framework to recognize and integrate the value of the psychological approach in the construction of processes linked to sustainable development. Accordingly, the psychology of sustainability and sustainable development could provide sustainable development processes for the employment of vulnerable workers. The contribution starts with the definition of the coordinates of a sustainable development process for vulnerable workers, anchoring it to the principles of decent work and inclusivity. Subsequently, positive variables involved in the sustainable development processes and their relationship with decent work and inclusivity are discussed. Moreover, positive healthy organizations are introduced as work environments capable to take care of the wellbeing, also, of vulnerable workers. Lastly, the applications of positive strength-based primary preventive interventions for vulnerable workers are analyzed. Perspectives for further research studies and interventions are also examined.

## Introduction

The 21st century is undergoing rapid processes of changes characterized by acceleration (Rosa, 2013; Rosa et al., 2017), “liquid” multifaceted features (Bauman, 2013), and globalizations (Sparks et al., 2001). These continuous changes have had an impact on the labor market with a drastic reduction in the number and quality of available jobs (Johnson et al., 2018; Blustein et al., 2019a,b). Thereby, the wellbeing of workers is particularly at risk (Robertson and Cooper, 2010; Cartwright and Cooper, 2014; Di Fabio and Kenny, 2016). Recent systemic shocks (e.g., September 11/2001, attacks in the United States; 2007–2008 the global financial crisis) and the COVID-19 pandemic have exacerbated these trends (Blustein et al., 2020; Kniffin et al., 2021). Subjects at higher risk are those who were already vulnerable, marginalized, and discriminated against (Blustein et al., 2020; Tamin et al., 2021). They are ethnic minorities, women, sexual minorities, immigrants, disabled people, precarious workers, young people, and older people, which are called by scholars as vulnerable workers (Blustein et al., 2020; Tamin et al., 2021). The concept of vulnerable workers is very wide, including a very heterogeneous variety of situations and conditions. In detail, we use the Psychology of Working Framework (PWF) as a critical perspective that is able to deeply reflect between stable and peripheral workers (Guichard, 2009), recognizing peripheral workers in terms of the unemployed, underemployed, immigrants, the poor, and people with disabling conditions (Blustein et al., 2014). According to this concept, vulnerable workers tend to be disproportionately excluded (Burgess and Connell, 2015) or even pushed out of the workforce (Guichard, 2009) since systemic imbalances in power and privilege discourage them from career paths (Blustein et al., 2014). As a result, workers from the vulnerable groups experience a progressive reduction in resources, becoming locked into a vicious circle that pushes them to the margin of the labor market (Fang and Gunderson, 2015). Such an employment gap leads to a reduction in opportunities for vulnerable workers to the point that finding a job is highly unlikely or extremely difficult (e.g., Blustein et al., 2019c; Andreassen et al., 2020; Schuring et al., 2020). To face this context, Blustein and colleagues have called upon vocational psychologists to advance new research studies and interventions to address vulnerable workers toward greater social inclusion and decent work (Guichard, 2009; Peiró and Tetrick, 2011; Di Fabio and Blustein, 2016; Blustein et al., 2019a,b; Di Fabio and Kenny, 2019a; Duffy et al., 2020).

Opportunities may stem from the psychology of sustainability and sustainable development, a current framework for research study and intervention (Di Fabio, 2017a,b; Di Fabio and Rosen, 2018). The psychology of sustainability and sustainable development integrates the value of the psychological science enriching the transdisciplinary field of sustainability science that contributes directly to United Nations (UN) 17 Sustainable Development Goals (United Nations, 2021). It is focused on advancing new processes connected to sustainability and sustainable development to improve human wellbeing and quality of life considering different kinds of environments (i.e., from nature to individuals, communities, organizations, societies, … until virtual environment and global environment) (Di Fabio and Rosen, 2018). The new keywords for managing developmental processes are promotion, enrichment, growth, and flexible change (Di Fabio and Rosen, 2018). This perspective analyzes sustainability *via* two axes of psychological reflection on what is sustainable for individuals, organizations, society, and the environment (Di Fabio, 2017a,b). The first axis (i.e., vertical) is related to the concept of time and involves dimensions of reflexivity that deals with understanding “where I come from,” establishes awareness of “where I am,” and proceeds to “where I will go” (Guichard, 2016; Di Fabio, 2017b). The second axis (i.e., horizontal) deals with the idea of space and others that are geographically near and far, and it is focused on dimensions of reflexivity that range from a self-centered position (i.e., egoistic) to a metacentric position (i.e., altruistic) (Maree, 2013; Di Fabio, 2017b). The reflexivity process on this axis is focused on mutual gain exploring the balance between gain for others and gain for the self (Di Fabio, 2017b; Di Fabio and Tsuda, 2018).

**FIGURE 1:**
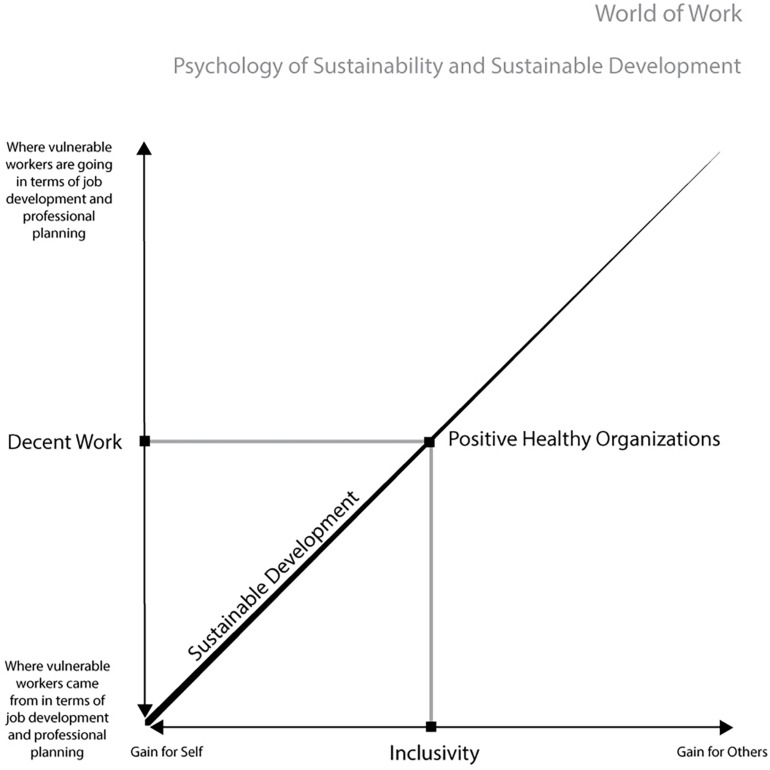
Coordinates of the sustainable development project for vulnerable workers.

Starting from these premises, coordinates for sustainable development process (Di Fabio, 2017b) in vulnerable workers could include on the vertical axis decent work (i.e., “where vulnerable workers are going” in terms of job development and professional planning) (Magnano et al., 2019, 2021; Zammitti et al., 2021) and on the horizontal axis inclusivity as a metacentric position to reduce the distance between vulnerable workers and the world of work. Moreover, sustainable development project for vulnerable workers has to consider optimal workplaces as able to protect the wellbeing of these individuals and to incorporate values in line with a sustainable development. According to this principle, advancing positive healthy organizations (Di Fabio, 2017a; Peiró, 2018) could be a promising solution. Positive healthy organizations are positive-oriented workplaces based on fostering resources, strengths, and talents of workers to achieve wellbeing (Di Fabio, 2017a,b; Johnson et al., 2018; Segura-Camacho et al., 2018; Peiró et al., 2020) including the new constructs of workplace relational civility (Di Fabio and Gori, 2016a) and sustainability of work-life projects (Di Fabio, 2017a; Di Fabio et al., 2018). Figure 1 illustrates the coordinates of our sustainable development project for vulnerable workers.

Thus, having in mind the idea of a sustainable development for vulnerable workers, which includes decent work and inclusivity as coordinates, this study aimed to discuss the following points: (a) examining positive psychological variables involved in sustainable development for vulnerable workers; (b) advancing the concept of positive healthy organization as workplaces capable to take care of the wellbeing of vulnerable workers; and (c) introducing the positive strength-based primary preventive perspective to foster wellbeing in vulnerable workers.

## Positive Psychological Variables for Sustainable Development in Vulnerable Workers

In this study, we examine the relationship between our coordinates for vulnerable workers (i.e., decent work and inclusivity) and positive variables involved in sustainable development. The positive variables are reflexivity, meaningfulness, connectedness, proximal zone, authentic self, the process of self-attunement, and harmonization (Di Fabio, 2017b; Di Fabio and Tsuda, 2018).

Concerning our purpose for a sustainable development project for vulnerable workers, decent work is the first coordinate on the vertical axis. This axis encompasses a process of psychological reflection investigating what is sustainable for vulnerable workers in their environments (from a macrolevel to a microlevel) (Di Fabio and Rosen, 2018). Decent work is defined by the psychology of working theory (PWT) as five job characteristics: (1) physically and psychologically safe working conditions, (2) adequate compensation, (3) sufficient rest/free time, (4) organizational values that incorporate family and social values, and (5) reasonable access to healthcare (Duffy et al., 2016).

From a macrolevel perspective (i.e., society), decent work for vulnerable workers represents a sustainable developmental goal in line with the United Nations (2021) and the International Labor Organization (International Labour Organization, 2020). Decent work agenda (International Labour Organization, 2008) indicates four objectives for the sustainable development of decent work: (1) creating a sustainable institutional/economic milieu; (2) advancing/enhancing proposals of social/labor protection that are sustainable and adapted to national circumstances; (3) promoting social dialogue and tripartism (i.e., close connections between governments, worker organizations, and employers); and (4) respecting, promoting, and realizing the fundamental human rights at work (International Labour Organization, 2008; pp. 9–11).

From a microlevel perspective (i.e., individual), decent work is characterized by the achievement of three arrays of basic human needs that, in turn, enable wellbeing and work fulfillment (job satisfaction and work meaning): (1) survival and power (i.e., essential resources for survival and adaptive levels of control over the needed resources); (2) social connections/contributions, (i.e., the need to connect and contribute to a larger community); and (3) self-determination (i.e., the need for human behaviors to align with authentic and meaningful goals) (Duffy et al., 2016; Blustein, 2019).

The sustainability of providing vulnerable workers with the means for surviving and power is in accordance with the call of ILO for “an adequate minimum wage” (International Labour Organization, 2020) and international literature studies (Carr et al., 2018). In this context, the minimum wage is a tool to protect vulnerable workers from unduly low wages while also reducing inequality *via* balanced wage policies (International Labour Organization, 2020). The satisfaction of needs of social connection at work for vulnerable groups is in line with the concept of connectedness, one of the core principles of sustainability from a psychological perspective. Psychology of sustainability and sustainable development has developed connectedness to energize sustainable structures by better balancing relationships with community, society, and the natural world (Di Fabio and Tsuda, 2018). The sustainability of self-determination and meaning at work for vulnerable groups is in line with meaningfulness, another psychological core principle of sustainability that makes it possible to support the coherence, direction, significance, and belonging of the sustainable construction (Schnell et al., 2013; Di Fabio, 2017b). Moreover, the achievement of the three arrays of basic human needs could represent the starting point to activate personal resources and enable the shape from a motivational to a meaning paradigm, enhancing the involvement of individuals and increasing the chances of success (Di Fabio and Blustein, 2016; Di Fabio and Kenny, 2016; Di Fabio, 2017b).

The second coordinate of our sustainable development project for vulnerable workers includes inclusivity (Blustein et al., 2019b) on the horizontal axis. Inclusivity is referred to all the efforts involved at various systemic levels for including individuals who have been marginalized by society (Blustein et al., 2019b). When inclusivity is under the light of sustainable development, it should be situated in the proximal zone of real progress (i.e., accounting for the real sustainability, not exceeding or investing less than available resources but always paying attention to peripheral workers with uncertain jobs and avoiding discrimination) (Guichard, 2009; Di Fabio, 2017b).

At the macrolevel (i.e., society), the proximal zone should be identified by national and international authorities, sustaining the efforts of vulnerable workers to access decent work both financially and with social recognition (Blustein et al., 2019a). At the microlevel (i.e., individual), the proximal zone for inclusivity should be assessed with the help of vocational psychologists applying sustainable career-management and self-management interventions for vulnerable workers (Blustein et al., 2019b).

To enhance inclusive psychological practice, PWT researchers have introduced the concept of agentic action (Blustein et al., 2019b). Agentic action encompasses three clusters of possible sources that assess how individuals and systems can change (Blustein et al., 2019c). The first cluster is critical reflection and action, which refers to identify the systemic causes of injustice through the analysis of the overt and covert aspects of society (Blustein et al., 2019c). The second cluster is proactive engagement, reflecting the role of work volition, proactive personality, and career adaptability, which provides the psychological resources and direction for individuals to manage their life and work (Blustein et al., 2019c). The third cluster is social support and community engagement that captures relational support and social contribution (Kenny et al., 2018; Blustein et al., 2019c).

From the psychology of sustainability and sustainable development perspective (Di Fabio, 2017b; Di Fabio and Rosen, 2018), each source of agentic action for vulnerable workers could be expanded by practitioners through reflexivity, harmonization, the authentic self, and the process of self-attunement (Di Fabio and Tsuda, 2018). Reflexivity enhances moving into a new perspective to observe the transition problem, clarify priorities, and envision a possible future to concretely build on through action (Di Fabio, 2017b; Di Fabio and Tsuda, 2018). It also considers the deep value of harmonization in order to gain authentic aims through the concept of balance (Di Fabio, 2017b; Di Fabio and Tsuda, 2018). The authentic self and the process of self-attunement individuate objectives that are most vital for the individual and community in line with the attainment of a life of true meaning (Di Fabio and Tsuda, 2018).

As a whole, a sustainable development project for vulnerable workers requires opening a transdisciplinary reflection space (Di Fabio and Rosen, 2018) and involving and connecting communities, international organizations, and local governments. Furthermore, the vocational psychologist could apply sustainable career-management and self-management processes (Blustein et al., 2019b), gaining psychological strengths, personal resources, and growth of vulnerable workers supporting them through the developmental process indicated by our coordinates.

## Enriching the Positive Perspective for Marginalized and Vulnerable Workers: Positive Healthy Organizations and Strength-Based Primary Prevention

Achieving decent work is the pillar of our sustainable development project for vulnerable workers. Moreover, the sustainability of the organizational environment (Di Fabio, 2017b) is another fundamental aspect to preserve over time the wellbeing of vulnerable workers. A growing number of scholars have discussed the beneficial role of the passage from ill-health to positive health in organizations (i.e., focusing on the talents of employees and gifts to attain enhanced performance, satisfaction, and wellness) (e.g., Schaufeli, 2004; Tetrick and Peiró, 2012). Similarly, the last two decades have seen a significant increase in interest in positive psychological approaches (Seligman and Csikszentmihalyi, 2000; Seligman, 2002) to promote the wellbeing of employees and organizational resources (i.e., studying excellence and success rather than weakness, failure, and damage) (Di Fabio and Kenny, 2016; Di Fabio, 2017a,b; Puigmitja et al., 2019). Also, the psychology of sustainability and sustainable development pointed out the “positive sustainability” (Di Fabio, 2017a; Di Fabio and Peiró, 2018). This innovative aspect extended the concept of regenerating resources to the enhancement of wellbeing advancing the new processes of “re-wellbeing, up-wellbeing, and crea(te)-wellbeing” (Di Fabio and Peiró, 2018). To this end, positive healthy organizations apply a positive primary prevention approach (Di Fabio, 2017b; Di Fabio et al., 2017; Di Fabio and Kenny, 2019b). Primary prevention is an early intervention that starts before the occurrence of problems (Caplan, 1964). It is centered on the building of the strengths of workers enhancing positive individual resources such as emotional intelligence (Di Fabio and Saklofske, 2014; Di Fabio, 2015), resilience (Di Fabio and Kenny, 2015; Wilson et al., 2017), and humor styles (Di Fabio and Duradoni, 2020). The focus on building resources and strengths (Di Fabio and Saklofske, 2021) in a primary preventive framework (Di Fabio and Kenny, 2016; Di Fabio et al., 2017), namely positive strength-based primary preventive, could be a promising approach for vulnerable workers.

An increasing number of studies have shown primary preventive approaches in the domain of sustainable sciences (e.g., Leporelli and Santi, 2019; Santi et al., 2019; Chotchoungchatchai et al., 2020; Nowacki et al., 2020), and scholars have advanced these interventions to foster the strengths of the employees (e.g., Di Fabio, 2019; Di Fabio and Duradoni, 2019, 2020). However, future studies are needed to expand knowledge on positive strength-based primary preventive interventions in vulnerable workers. As said, a reflection on the efficacy of these latter interventions in vulnerable workers deserves particular attention.

First, outside the concept of positive healthy organizations, positive strength-based primary preventive interventions could have limited efficacy. In fact, positive healthy organizations in a preventive perspective are centered on the enhancement of strengths at all organizational levels (i.e., the individual, the group, the organization, and the inter-organizational level) (Henry, 2005; Di Fabio, 2017a,b). Therefore, psychological resources are maintained through the right balance of a particular situation, sector, and culture, allowing endurance and sustainability of wellbeing (Di Fabio, 2017a,b; Di Fabio and Rosen, 2018). Such characteristics could be particularly optimal for vulnerable workers, which are in need of more complex interventions to be incentivized in the workplace (e.g., Srivastava and Chamberlain, 2005; Truxillo et al., 2012; Di Fabio and Gori, 2016a; Di Fabio and Duradoni, 2019).

Second, positive strength-based primary preventive interventions implemented without considering the sustainability of the work-life project (Di Fabio, 2017a,b) could have poor efficacy. Sustainability of the work-life project is fundamental in the employment of vulnerable workers (Stoilova et al., 2020; Opute et al., 2021) to find a balance between caregiving roles, family values, and working roles (Núñez et al., 2020; Rodríguez-Rivero et al., 2020). Conversely, the failure in work-life balance could have detrimental effects on the health of vulnerable workers (Blustein et al., 2019a,b). Positive healthy organizations link the sustainability of life-work projects to meaningful construction in employees *via* the positive psychological narratives of meaning (Di Fabio, 2014b; Di Fabio and Maree, 2016) that are a key part of the primary preventive approach. Positive psychological narratives of meaning are based on the intervention of Savickas “stored self” (Savickas, 2011) and help employees to build and foster optimal stories, beginning with specific real-life work circumstances (i.e., “from facts”) and concluding with attention on relationships and details of meaning (“from perception of the facts” and “from success experience,” respectively) (Blustein, 2011; Di Fabio and Blustein, 2016).

Third, positive strength-based primary preventive interventions on vulnerable workers that neglect the relational aspects of work could have a poor outcome, too. Relational aspects in the workplace are threatened for vulnerable workers, being at risk of various forms of discrimination, for example, racism (e.g., Storer et al., 2020; Rosander and Blomberg, 2021), sexism (e.g., Crewe and Wang, 2018; Velez et al., 2018), and exclusion (Cox, 2020; Friedman and Rizzolo, 2020). To prevent these phenomena, positive healthy organizations comprise workplace relational civility (i.e., relational decency, relational culture, and relational readiness) to strengthen positive interactions in organizational groups and reduce conflict in organizations (Di Fabio and Gori, 2016a).

Lastly, it is important to consider forms of leadership able to promote positive sustainability for vulnerable workers (Di Fabio and Peiró, 2018). The positive healthy organizational framework has recently developed the new construct of human capital sustainability leadership that integrates sustainability leadership (Di Fabio and Peiró, 2018) with ethical leadership (Yukl et al., 2013), mindful leadership (Herold, 2013), and servant leadership (Greenleaf, 1977). Sustainability leadership concerns the use of vigilant decision-making processes and sustainable development of human resources through continuous learning processes (Di Fabio and Peiró, 2018). Ethical leadership encompasses fairness and aims to empower members of an organization (Yukl et al., 2013). Mindful leadership refers to paying attention to the present moment, recognizing and controlling personal cognition, and being aware of the own presence of an individual and the influence on other people (Herold, 2013). Servant leadership consists of sharing moral responsibility, allowing the growth of followers for their interests and needs and not only for the interests of the organizations (Greenleaf, 1977).

## Conclusion

The psychology of sustainability and sustainable development (Di Fabio, 2017a,b; Di Fabio and Rosen, 2018) could be the framework for designing and constructing a sustainable project for long-lasting employment of vulnerable groups, which encompasses decent work and inclusivity as coordinates (Blustein et al., 2019a). Positive interventions from a microlevel to a macrolevel are available to ensure the sustainability of the project coordinates. The sustainability of decent work is enriched through metacentric reflexivity, connectedness, and meaningfulness (Di Fabio and Blustein, 2016; Duffy et al., 2016; Di Fabio, 2017a,b). Sustainability of inclusivity is fostered *via* reflexivity, harmonization, the authentic self, and the process of self-attunement (Di Fabio, 2014b, 2017a,b; Di Fabio and Tsuda, 2018). Positive healthy organizations (Di Fabio and Peiró, 2018; Di Fabio et al., 2020) are workplaces capable to take care of the wellbeing of vulnerable workers by applying positive strength-based primary preventive interventions (Di Fabio and Kenny, 2016; Di Fabio and Saklofske, 2021) at all the organizational levels. For example, the construction of strengths at the individual level encompasses three types of interventions: intrapreneurial self-capital (Di Fabio, 2014a), acceptance of the change (Di Fabio and Gori, 2016b), and life project reflexivity (Di Fabio et al., 2018). Intrapreneurial self-capital intervention deals with the frequent career challenges by creating innovative solutions and turning the constraints of the environment into resources (Di Fabio, 2014a). Acceptance of change intervention referred to the tendency to embrace rather than shy away from changes since acceptance is viewed as positive for the wellbeing of an individual (Di Fabio and Gori, 2016b). Life projectuality intervention is aimed to stimulate the reflexivity of an individual regarding their future work-life projects by analyzing three areas: projectuality, authenticity, and acquiescence (Di Fabio et al., 2018). Furthermore, positive healthy organizations further protect and sustain endurance of wellbeing for vulnerable workers *via* the sustainability of work-life balance (Haar et al., 2014; Di Fabio, 2017a,b), workplace rational civility (Di Fabio and Gori, 2016a), and human capital sustainability leadership (Di Fabio and Peiró, 2018).

Concerning future research hypotheses, a recent study has provided new insights into the role of job crafting to shape decent work in vulnerable workers (Svicher and Di Fabio, 2021). Consequently, future research studies for vulnerable workers could also explore the links between job crafting, decent work, the psychology of sustainable development principles, and the healthy organization milieu. Another line of research study could investigate the relationship between decent work and occupational fatigue in vulnerable workers, studying the contribution of decent work in protecting vulnerable workers from occupational fatigue and the possible adjunctive improvement obtained by applying positive strength-based primary preventive interventions.

In brief, the psychology of sustainability and sustainable development represents an opportunity to create new research studies and intervention strategies to advance decent work as a human right (Duffy et al., 2017; Blustein et al., 2019c). This challenging opportunity stems from the virtuous circle provided by linking together sustainability science, social justice policies (International Labour Organization, 2020; United Nations, 2021), positive psychological processes revitalizing strengths (e.g., Di Fabio, 2017a; Di Fabio and Saklofske, 2014, 2021; Di Fabio and Blustein, 2016; Di Fabio and Kenny, 2016), and positive healthy organizations protecting human dignity and equality (Di Fabio, 2017b; Di Fabio and Peiró, 2018).

## Data Availability Statement

The original contributions presented in the study are included in the article/supplementary material, further inquiries can be directed to the corresponding author.

## Author Contributions

AS wrote the first draft of the manuscript. ADF conceptualized the manuscript, supervised and tutored AS, reviewed, edited, and wrote the final draft of the manuscript. Both authors contributed to the article and approved the submitted version.

## Conflict of Interest

The authors declare that the research was conducted in the absence of any commercial or financial relationships that could be construed as a potential conflict of interest.

## Publisher’s Note

All claims expressed in this article are solely those of the authors and do not necessarily represent those of their affiliated organizations, or those of the publisher, the editors and the reviewers. Any product that may be evaluated in this article, or claim that may be made by its manufacturer, is not guaranteed or endorsed by the publisher.
